# Nanomaterial-Modified Thermally Expandable Thixotropic Gel for Enhanced Plugging Performance in High-Temperature Fractured Formations

**DOI:** 10.3390/gels12090813

**Published:** 2026-09-05

**Authors:** Dong Liu, Xian Zhu, Xiangwei Cai, Gang Chen, Xu Luo, Wenjun Shan

**Affiliations:** 1 Natural Resources Survey Institute of Hunan Province, Changsha 410007, China; 2 Oil and Gas Survey, China Geological Survey, Beijing 100083, China

**Keywords:** thermally expandable gel, nanomaterial modification, fracture plugging, shear recovery, thixotropy

## Abstract

Lost circulation in fractured formations remains a critical challenge in petroleum engineering, significantly hindering drilling efficiency and increasing operational risks. To overcome the limitations of conventional plugging materials under high-temperature and complex fracture conditions, a nanomaterial L-modified thermally expandable thixotropic gel system was developed. By incorporating a nanomaterial with strong thixotropic characteristics and multifunctional monomers, multiscale network regulation and interfacial enhancement were achieved. Systematic rheological and performance evaluations were conducted to optimize the formulation, enabling a balanced combination of flowability, structural recovery, and plugging performance. Experimental results demonstrate that the gel exhibits pronounced shear-thinning behavior and excellent thixotropic recovery, ensuring superior injectability under high shear and strong structural integrity under low-shear conditions. The system also maintains good thermal stability below 150 °C and achieves effective plugging across varying fracture scales. In addition, moderate salinity enhances network strength, whereas excessive salt concentration weakens structural stability and recovery capability. This study provides a novel design strategy for nanomaterial-regulated thermally expandable gels and offers a promising solution for lost circulation control in complex fractured formations.

## 1. Introduction

In petroleum engineering, lost circulation remains one of the most critical technical challenges encountered during drilling operations. It not only results in substantial drilling fluid losses and increased operational costs but may also trigger wellbore instability, blowouts, and other complex downhole incidents, thereby increasing non-productive time and posing risks to reservoir integrity and environmental safety [1,2,3,4]. Statistical analyses indicate that nearly 50% of fluid losses in global oil and gas operations are attributed to formation losses, leading to economic losses of billions of dollars annually [5]. Moreover, fracture apertures are highly sensitive to temperature and pressure variations, exhibiting a wide and multiscale distribution. Conventional bridging materials suffer from limited size compatibility, making them ineffective in sealing complex fracture networks and often resulting in recurrent fluid losses [6]. With the progression of oil and gas exploration toward deep and ultra-deep reservoirs, conventional plugging materials exhibit insufficient adaptability under high-temperature and complex fracture conditions. Therefore, the development of innovative plugging materials with both high-temperature resistance and thermal expansion capability is of great engineering significance for effective lost circulation control in complex formations [7,8,9].

To mitigate lost circulation, plugging treatments are commonly employed to construct load-bearing sealing layers within the wellbore wall or loss channels, thereby reducing the loss rate and restoring wellbore integrity. As the key medium in lost circulation control, the composition, morphology, and action mechanism of plugging materials directly determine their sealing efficiency and field applicability. According to their plugging mechanisms and material forms, existing lost circulation materials can generally be classified into bridging materials, high-fluid-loss materials, curable materials, fluid-absorbing and swelling materials, and polymer gels [10,11]. These materials differ substantially in particle-size matching, rheological behavior, plugging strength, deformability, response rate, and placement strategy, enabling their application to loss channels with different dimensions and under diverse formation conditions. Although these materials have demonstrated effectiveness under various lost circulation conditions, their applicability in complex deep formations remains constrained by their intrinsic structures and plugging mechanisms. Bridging materials primarily rely on particle-size matching with loss channels, making them highly sensitive to variations in fracture dimensions and limiting their ability to form continuous and compact sealing structures within complex fracture networks [12]. High-fluid-loss materials form filter cakes or dense sealing layers through rapid fluid loss; however, the resulting structures are often relatively loose and exhibit limited long-term pressure-bearing capacity [13]. Curable materials can provide high ultimate sealing strength, but their curing kinetics are difficult to precisely regulate, potentially resulting in premature curing, pumping difficulties, or incomplete sealing. Fluid-absorbing and swelling materials can fill loss spaces through water uptake and expansion, whereas their response rate and swelling degree are strongly affected by temperature, salinity, and the surrounding fluid environment [14]. In contrast, polymer gels possess continuous three-dimensional network structures and can adapt to irregular pores and fractures through the synergistic effects of viscoelastic deformation, fluid absorption, swelling, and interfacial adhesion, making them particularly promising for sealing complex loss channels.

Currently, most lost circulation gels are constructed based on acrylamide and acrylic acid systems, including acrylic resin-based gels and self-healing hydrophobically associative gels. These materials have been widely utilized due to their favorable thermal stability, ease of grafting or crosslinking, and excellent water absorption and swelling capacity [15,16,17,18]. In addition, the incorporation of hydrophobically associative structures significantly enhances viscoelasticity, enabling the development of gels with both thermo-responsive and self-healing capabilities [19]. Zhong et al. [20] developed an OAP plugging material that exhibits effective fracture sealing at high concentrations; however, it significantly increases the viscosity of drilling fluids, leading to potential adverse effects. He et al. [21] prepared a self-healing hydrophobically associative polyacrylamide hydrogel capable of structural recovery at 70 °C through hydrophobic interactions. Sun et al. [22] reported that bridging-type materials are mainly applicable to permeable formations or mild losses, but their poor size matching and limited thermal stability result in low pressure-bearing capacity. Loukili et al. [23,24] demonstrated that cationic polyacrylamide undergoes degradation at elevated temperatures, impairing its flocculation performance and indicating limited thermal stability. Consequently, conventional polymers still face inherent limitations when applied in deep and complex formations. On one hand, low-molecular-weight components exhibit limited functionality at high temperatures; on the other hand, high-molecular-weight polymers are prone to degradation under high shear. More importantly, conventional polymers exhibit limited functionality and often require combination with materials such as asphalt or rigid nanoparticles, resulting in poor adaptability to complex pore–fracture systems and inefficient plugging performance. Therefore, for complex deep and ultra-deep formations, an ideal polymer-gel plugging material should combine good injectivity with high low-shear viscosity, gel strength, and viscoelasticity, thereby enabling the formation of a stable sealing structure within loss channels. Meanwhile, pronounced shear-thinning behavior is desirable to reduce flow resistance during high-shear pumping, while subsequent viscosity recovery and gel-structure formation can enhance material retention within the loss channels. Considering the high-temperature and high-salinity conditions encountered in deep formations, the gel should also possess sufficient thermal stability, salt tolerance, and water-absorption and swelling capacity to accommodate complex pore–fracture structures and maintain an effective sealing volume.

Based on these requirements, incorporating nanomaterials and regulating the polymer-gel network structure provide an effective approach for improving the overall plugging performance of gels. Nanomaterials can interact with polymer chains through multiple physical interactions and act as reinforcing components within the network, thereby enhancing the mechanical strength, viscoelasticity, and structural stability of the gel. By rationally adjusting the nanomaterial content, a balance between injectivity and plugging capacity can be achieved, allowing the gel to be readily pumped during treatment while subsequently forming a highly load-bearing gel structure within the loss channel. Therefore, developing a nanocomposite gel that integrates shear-thinning behavior, high gel strength, favorable thixotropic recovery, thermal and salt stability, and water-absorption and swelling capacity is of considerable significance for improving lost circulation control in complex deep formations. In recent years, nanomaterials have offered new avenues for improving drilling fluid performance due to their high specific surface area and unique interfacial effects [25]. Among them, polymer nanogels, as crosslinked soft nanoparticles with three-dimensional network structures, have demonstrated considerable application potential [26,27]. Compared with linear polymers, the network architecture of nanogels effectively suppresses irreversible chain disentanglement and degradation, resulting in superior thermal stability and shear resistance. Their three-dimensional networks interact with both solid and liquid phases through physical entanglement and chemical crosslinking, forming a stable spatial network that significantly enhances viscosity and yield strength [28,29,30]. Furthermore, rational monomer design can impart specific functionalities, thereby enhancing adsorption capacity and hydration inhibition [31]. Therefore, the integration of nanomaterials with gel systems represents a promising multifunctional strategy for addressing drilling challenges in deep and complex formations.

Based on the above considerations, a thermally expandable nanogel system is proposed in this study. A nanomaterial (denoted as L) with strong thixotropic characteristics was incorporated to regulate the thermal expansion behavior of the gel system. Under shear, the gel rapidly transitions from a solid-like to a fluid-like state, exhibiting excellent injectability. Upon removal of shear, the system quickly recovers into a stable three-dimensional network, enabling efficient sealing of loss channels. In addition, three functional monomers were introduced to enhance interfacial interactions and hydrophobic associations, thereby achieving efficient thermal expansion while improving network stability.

## 2. Results and Discussion

### 2.1. Rheological Properties

#### 2.1.1. Shear-Thinning Behavior

**Figure 1:**
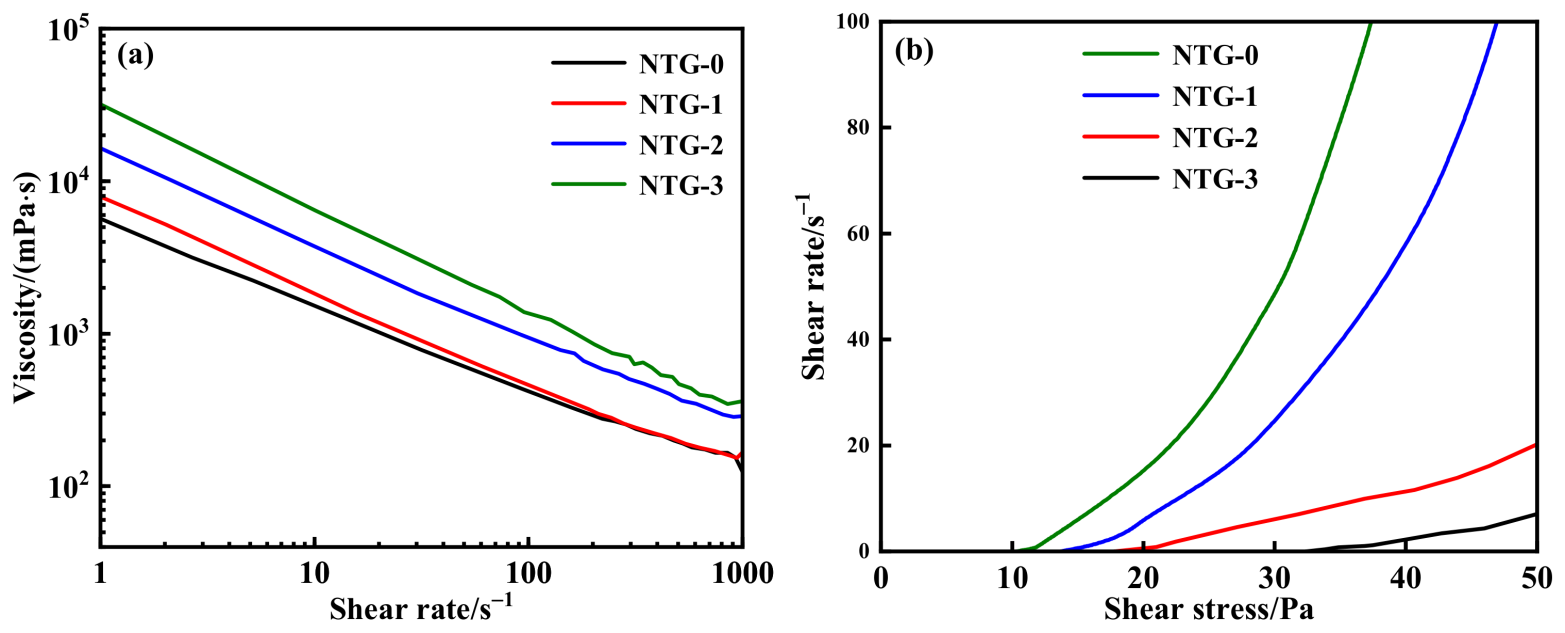
Shear-thinning property (**a**) and yield stress (**b**) curves of gel system.

Following the synthetic procedure described in Section 4.2, NTG gels containing different concentrations of modified Laponite nanomaterial L were prepared, and their rheological properties were subsequently evaluated. As shown in Figure 1a, the viscosity of all gel systems decreased with increasing shear rate, exhibiting typical shear-thinning behavior, which indicates favorable pumpability. The NTG-0 sample without nanomaterial L exhibited the lowest viscosity, with values of 124 mPa·s at 1000 s^−1^ and 5663 mPa·s at 1 s^−1^. With increasing nanomaterial L content, the viscosity of the system increased significantly. In particular, NTG-3 exhibited viscosities of 362 mPa·s and 31,942 mPa·s at high and low shear rates, respectively, indicating enhanced shear-thinning behavior. As shown in Figure 1b, the yield stress increased with increasing nanomaterial L content. At 3 wt% loading, the yield stress increased by 212.5% compared to NTG-0 (from 10.35 Pa to 32.34 Pa). These results demonstrate that the incorporation of nanomaterial L significantly enhances the shear resistance and structural stability of the gel.

These results demonstrate that the incorporation of Modified nano-magnesium lithium silicate material L significantly enhances the shear resistance and structural stability of the gel. The observed enhancement can be attributed to the synergistic reinforcement effect of nanomaterial L. On one hand, the abundant hydroxyl groups on the surface of nanomaterial L can form multiple non-covalent interactions, including hydrogen bonding, electrostatic interactions, and van der Waals forces, with functional groups such as amide and quaternary ammonium groups along the polymer chains. On the other hand, its high specific surface area and layered structure facilitate the formation of a more compact three-dimensional network, thereby enhancing the cohesion of the system, as reflected by increased viscosity and yield stress. To quantitatively describe the rheological behavior, the Herschel–Bulkley model was employed for fitting (Table 1), yielding correlation coefficients (R^2^) greater than 0.97, indicating excellent agreement between the model and experimental data. The yield stress (τ_0_) increased from 12.66 Pa to 30.23 Pa with increasing nanomaterial content, consistent with experimental observations. The consistency index (K) increased from 0.61 to 14.23, suggesting that the incorporation of nanomaterial L significantly enhanced the crosslinking density of the network structure, thereby improving its structural robustness. The flow behavior index (n) decreased from 0.70 to 0.45, indicating strengthened shear-thinning behavior and improved flow adaptability.

#### 2.1.2. Viscoelasticity and Thixotropic Recovery Behavior

To comprehensively evaluate the structural robustness and recovery capability of the gel systems, their viscoelastic and thixotropic behaviors were systematically investigated.

**Figure 2:**
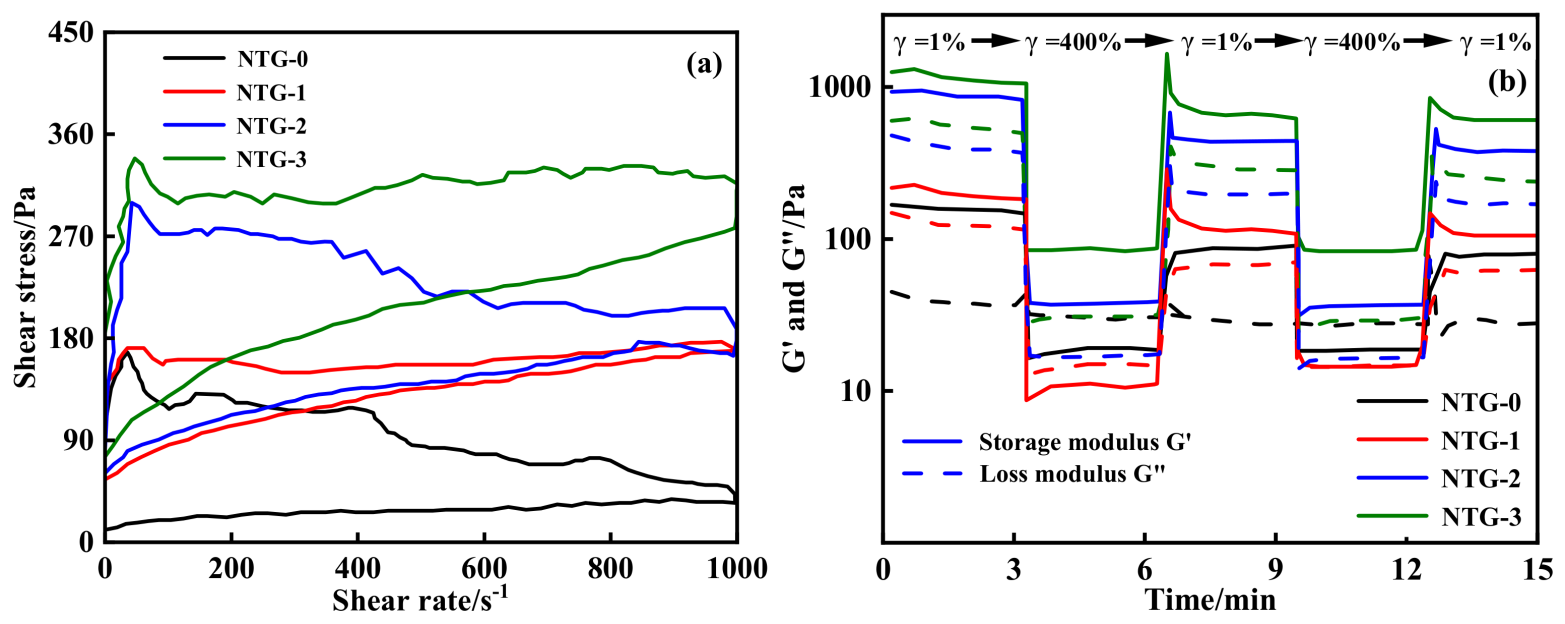
Thixotropy test of gel systems: (**a**) thixotropy loop curve; (**b**) multistage low strain–high strain shear recovery curve.

Thixotropy is a critical rheological parameter governing post-injection structural recovery and plugging stability. To characterize the structural breakdown and recovery under shear perturbation, thixotropic loop tests and step-strain measurements were conducted, as shown in Figure 2. In the thixotropic loop tests, the shear rate was cyclically varied between 1 s^−1^ and 1000 s^−1^ to probe the thixotropic response of the gels. As shown in Figure 2a, all samples exhibited pronounced hysteresis loops, where the downward curves were lower than the upward curves, indicating a time-dependent lag between structural breakdown and recovery during shear. With increasing nanomaterial L content, the overall shear stress increased, suggesting enhanced structural strength. Notably, NTG-3 exhibited a maximum shear stress of approximately 332 Pa. The hysteresis loop areas were calculated as 67,143 Pa·s^−1^ (NTG-0), 33,852 Pa·s^−1^ (NTG-1), 78,422 Pa·s^−1^ (NTG-2), and 121,106 Pa·s^−1^ (NTG-3), demonstrating that the incorporation of nanomaterial L significantly enhances the thixotropic behavior. This behavior can be attributed to the multiple physical interactions between nanomaterial L and polymer chains, which reinforce the network structure. Under high shear, the network undergoes temporary disruption, followed by gradual recovery once the shear is removed. To quantitatively assess shear recovery, multistep strain tests were further conducted, as presented in Figure 2b. During the low–high–low strain cycles, all samples exhibited characteristic structural breakdown and recovery in their viscoelastic moduli. Upon exposure to high strain (400%) for 3 min, the storage modulus (G′) dropped sharply below the loss modulus (G″), indicating disruption of the gel network and transition to liquid-like behavior. When the strain was reduced back to 1%, G′ rapidly recovered. After two shear cycles, the recovery ratio exceeded 50%, with NTG-2 and NTG-3 maintaining G′ values above 500 Pa, indicating strong structural recovery and mechanical integrity. In addition, the initial G′ values varied among samples, with NTG-2 and NTG-3 exceeding 1000 Pa, significantly higher than that of NTG-0 (180 Pa), confirming that nanomaterial L effectively enhances network strength and elasticity.

A comparison between NTG-2 and NTG-3 revealed that further increasing the nanomaterial content did not significantly enhance the viscoelastic moduli, while the consistency index increased markedly, which may hinder field application. Considering comprehensive factors, NTG-2 was ultimately selected as the optimal thixotropic gel system for subsequent evaluations of thermal stability, salt tolerance, and plugging performance.

### 2.2. Gelation Behavior and Mechanical Strength

**Figure 3:**
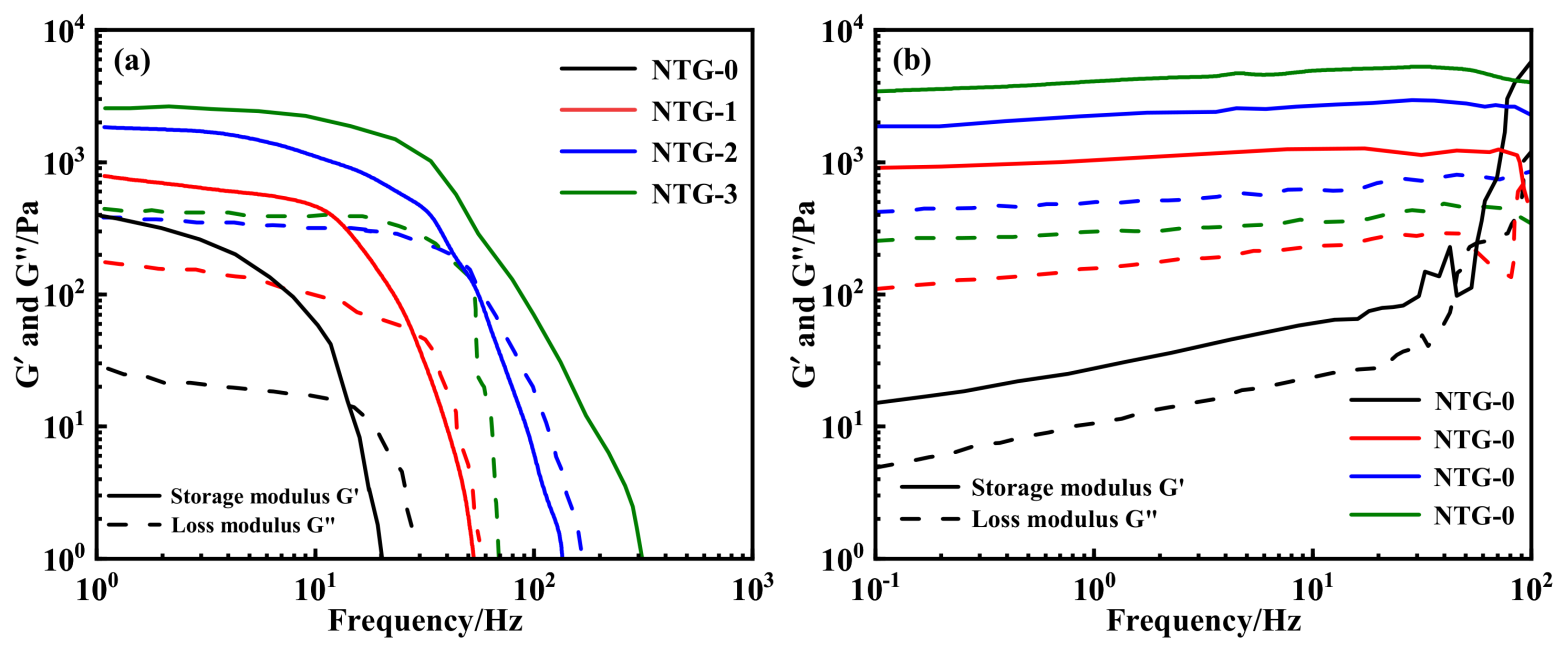
Viscoelastic curve of gel system: (**a**) Stress scan; (**b**) Frequency scan.

Furthermore, viscoelastic measurements were conducted using a Haake rheometer to quantitatively assess gel strength, as shown in Figure 3. In the stress sweep test (Figure 3a), the storage modulus (G′) of all samples was significantly higher than the loss modulus (G″) at low shear stress, indicating elastic-dominant behavior. As the shear stress increased, G′ gradually decreased and intersected with G″ at a critical stress, signifying the disruption of the gel network and a transition from solid-like to liquid-like behavior. Among all samples, NTG-2 exhibited the highest critical stress (75 Pa), suggesting that higher nanomaterial L content enhances shear stability and structural integrity. Based on the linear viscoelastic region obtained from the stress sweep, the frequency sweep was conducted at a constant stress of 1 Pa. As shown in Figure 3b, G′ and G″ of NTG-0 were comparable at low frequencies, indicating weak gel behavior. In contrast, NTG-1, NTG-2, and NTG-3 exhibited significantly higher G′ values across the entire frequency range, stabilizing at approximately 920 Pa, 2748 Pa, and 4622 Pa, respectively, far exceeding that of NTG-0 (21 Pa). Moreover, G′ consistently remained higher than G″, confirming the formation of strong gel networks.

### 2.3. Thermal Stability and Expansion Behavior

**Figure 4:**
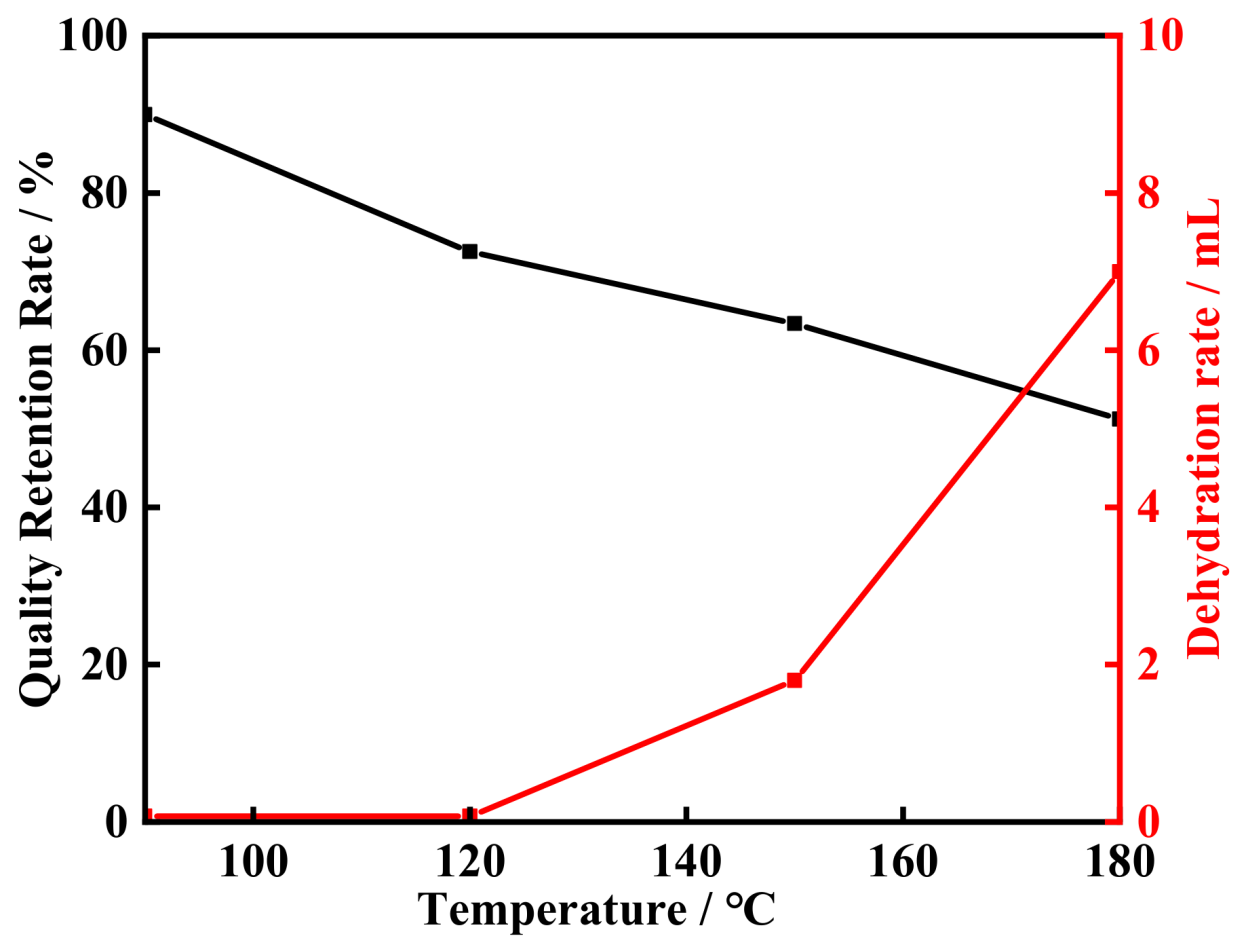
Mass retention and dehydration of gel system after aging at different temperatures.

**Figure 5:**
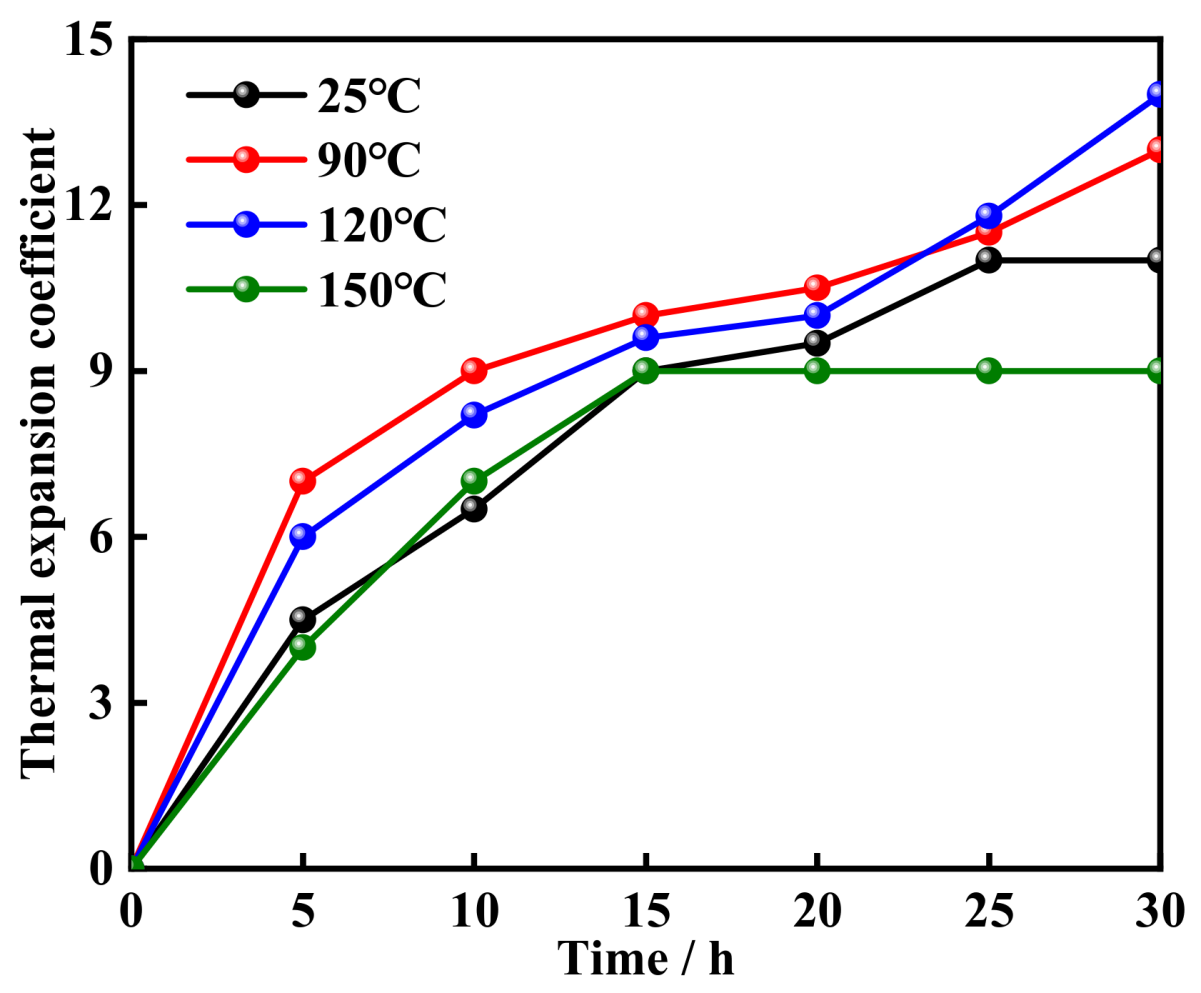
Expansion coefficient of gel system NTG-2 at different temperatures.

The thermal stability and swelling behavior of NTG-2 at different temperatures are presented in Figure 4 and Figure 5. The volume change of NTG-2 is primarily associated with water uptake and retention within the gel network. Hydrophilic groups within the three-dimensional polymer network interact with water molecules through hydrogen bonding and hydration, facilitating water penetration into the network and inducing network expansion, thereby producing pronounced water-swelling behavior. Temperature significantly influences water uptake and volume variation by regulating polymer-chain mobility, the hydration of hydrophilic groups, and water transport within the network.

After aging at 90, 120, and 150 °C for 24 h, NTG-2 exhibited high mass retention (>60%) and low dehydration (<2 mL), with no significant variation in gel strength, indicating that the gel network maintained good structural integrity below 150 °C. With increasing temperature, enhanced polymer-chain mobility and water transport within the network, together with strengthened interactions between hydrophilic groups such as amide groups and water molecules, facilitated water penetration into the gel matrix. Consequently, increasing temperature within a moderate range promoted water uptake and swelling of the gel.

NTG-2 exhibited a pronounced temperature-dependent swelling behavior. From room temperature to 120 °C, the swelling capacity progressively increased and reached a maximum swelling ratio of 14 at 120 °C, followed by a decrease to 9 at 150 °C. This variation was mainly associated with the water-uptake capacity of the gel network and its water-retention ability at elevated temperatures. Within an appropriate temperature range, increasing temperature promoted the hydration of hydrophilic groups and water diffusion, allowing more water to enter the network and drive volumetric expansion. At higher temperatures, however, the stability of the network decreased and some intermolecular interactions were weakened, while the enhanced thermal motion of water molecules facilitated water release from the network, resulting in a reduced swelling ratio. After aging at 90 and 120 °C for 24 h, the mass retention of NTG-2 was 89% and 72%, respectively, while dehydration was negligible below 120 °C, indicating good water-retention capacity and structural stability under moderately elevated temperatures. As the aging temperature was further increased to 150 °C, the gel system still maintained relatively good structural stability, indicating that temperatures up to 150 °C generally remained within the stable range of the NTG-2 network structure and water-retention capability. When the temperature was further increased beyond 150 °C to 180 °C, the mass retention of NTG-2 decreased markedly to 51%, accompanied by an increase in dehydration to 7 mL, indicating pronounced water release and partial solid–liquid separation. Compared with the relatively stable state observed at temperatures up to 150 °C, the gel system exhibited a substantial deterioration in both mass retention and water-retention capability above 150 °C, indicating that 150 °C represents a critical temperature point at which a pronounced transition in the thermal stability of NTG-2 occurs. Although pronounced dehydration occurred at 180 °C, the solid gel still retained relatively high strength, indicating that its initial network framework was not completely disrupted. At elevated temperatures, continuous water release from the gel network induced dehydration and volumetric contraction, while the reduced stability of the network further impaired its water-retention capability. Thus, NTG-2 exhibited a pronounced temperature-dependent response upon heating: below 150 °C, the gel network maintained relatively good water retention and structural stability, whereas above 150 °C, water release and network destabilization gradually became dominant, driving the transition from a water-swollen state to a dehydration-induced contracted state.

The incorporation of nanomaterial L further enhanced the structural stability and water-retention capacity of the gel network. Physical entanglement and multipoint interactions between the L nanosheets and polymer chains increased the connectivity of the network and restricted excessive polymer-chain mobility, thereby facilitating the preservation of network integrity and water distribution within the gel matrix. This reinforcing effect contributed to the high water-swelling capacity of NTG-2 within the appropriate temperature range and enabled the gel to retain considerable structural strength under elevated temperatures.

### 2.4. Salt Tolerance

**Figure 6:**
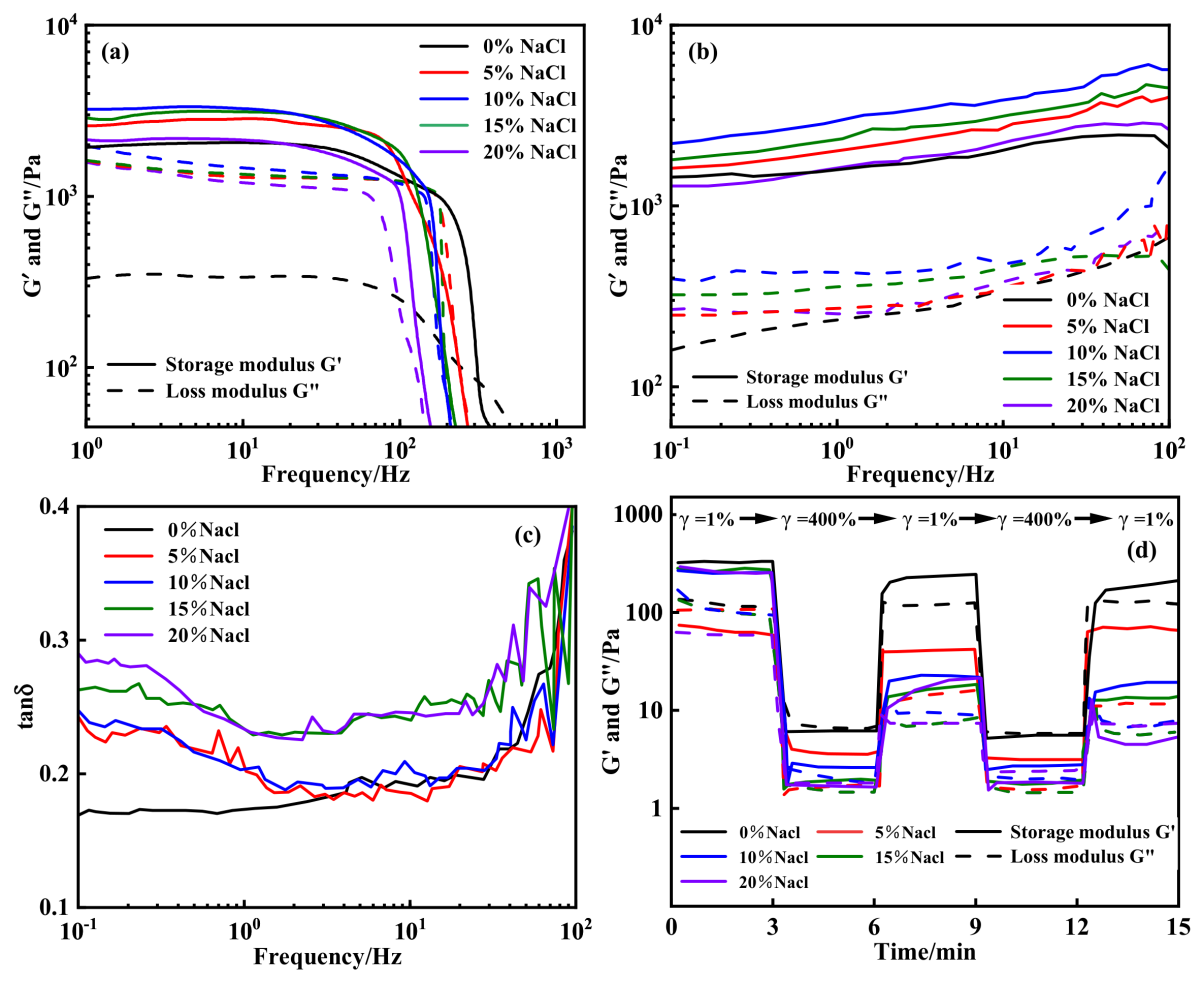
Actual vs. predicted scatter plot. Viscoelasticity and recovery test of gel system and different concentrations of NaCl: (**a**) Stress scan; (**b**) Frequency scan; (**c**) Tan 8 of frequency scan; (**d**) Multistage shear recovery.

Gel systems were prepared using NaCl solutions with varying mass fractions, and their viscoelastic moduli and cyclic shear recovery were measured using a Haake rheometer to evaluate salt tolerance. As shown in Figure 6, stress sweep results indicate that the storage modulus (G′) remained higher than the loss modulus (G″) under all salinity conditions, demonstrating solid-like behavior with elastic deformation capability. Both G′ and G″ remained stable within the stress range of 1–100 Pa, indicating the linear viscoelastic region; thus, 10 Pa was selected as the constant stress for frequency sweep tests. Frequency sweep results show that G′ increased as the NaCl concentration rose from 0% (black line) to 10% (blue line), suggesting that Na^+^ effectively screens electrostatic repulsion between polymer chains, promoting closer chain packing and formation of a denser network (G′ ≈ 2000 Pa). However, when the salt concentration increased from 10 wt% to 20 wt% (purple line), G′ gradually decreased, likely due to disruption of hydrogen bonding and other non-covalent interactions by excessive ions, resulting in a looser network and reduced stability (G′ < 1000 Pa). Furthermore, the loss factor (tanδ = G″/G′) indicates that gels formed under salt-free or low-salinity (10 wt%) conditions exhibit low tanδ values (<0.3), reflecting strong elasticity. In contrast, higher salinity leads to increased tanδ values, indicating weakened elastic behavior. Alternating strain tests (1–400%) revealed shear recovery behavior. Gels prepared with deionized water exhibited good recovery after 3 min of shear, with recovery ratios of 65.78% and 49.66% after two cycles. In contrast, gels prepared with brine showed significantly reduced recovery, with the lowest values observed at 20 wt% NaCl (4.23% and 1.09%). This behavior can be attributed to the strong electrostatic screening effect of high salt concentrations, which restricts polymer-chain mobility and hinders network reconstruction after shear, thereby prolonging recovery time. In summary, moderate Na^+^ concentrations enhance network crosslinking, whereas excessive salinity (>10 wt%) weakens structural strength and significantly reduces post-shear recovery capability.

### 2.5. Plugging Performance

**Figure 7:**
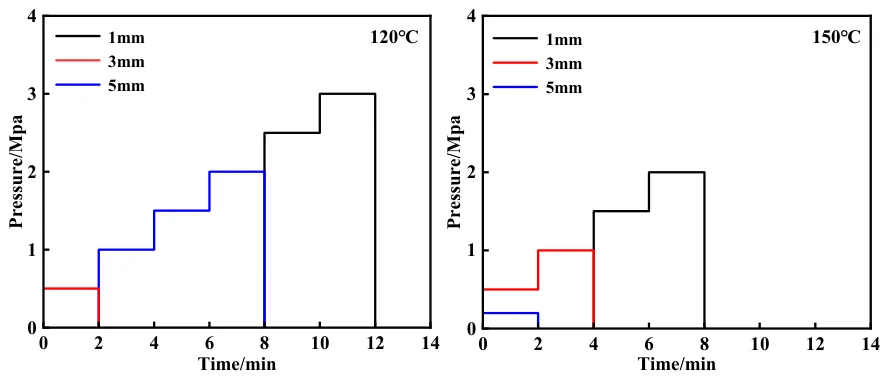
Breakthrough pressure of gel in cracks of different widths at different temperatures.

To better simulate real lost circulation conditions, the plugging performance of the thixotropic gel was evaluated by measuring the breakthrough pressure in artificial fractured cores with different fracture widths using a core flooding apparatus. The breakthrough pressures of NTG-2 were measured at 120 °C and 150 °C for fracture widths of 1 mm, 3 mm, and 5 mm, as shown in Figure 7. At 120 °C, the breakthrough pressures were 3 MPa, 2 MPa, and 0.5 MPa for fracture widths of 1 mm, 3 mm, and 5 mm, respectively. At 150 °C, the breakthrough pressures decreased to 2 MPa and 1 MPa for 1 mm and 3 mm fractures, respectively, and further dropped to 0.2 MPa for 5 mm fractures. With increasing fracture width, the required filling volume increases while flow resistance decreases, making it difficult to form a dense plugging layer, thereby reducing the breakthrough pressure. Elevated temperature further weakens the three-dimensional network by reducing non-covalent interactions, leading to lower crosslink density, increased fluidity, and consequently diminished plugging performance.

### 2.6. Comparison of Sealing Performance

**Figure 8:**
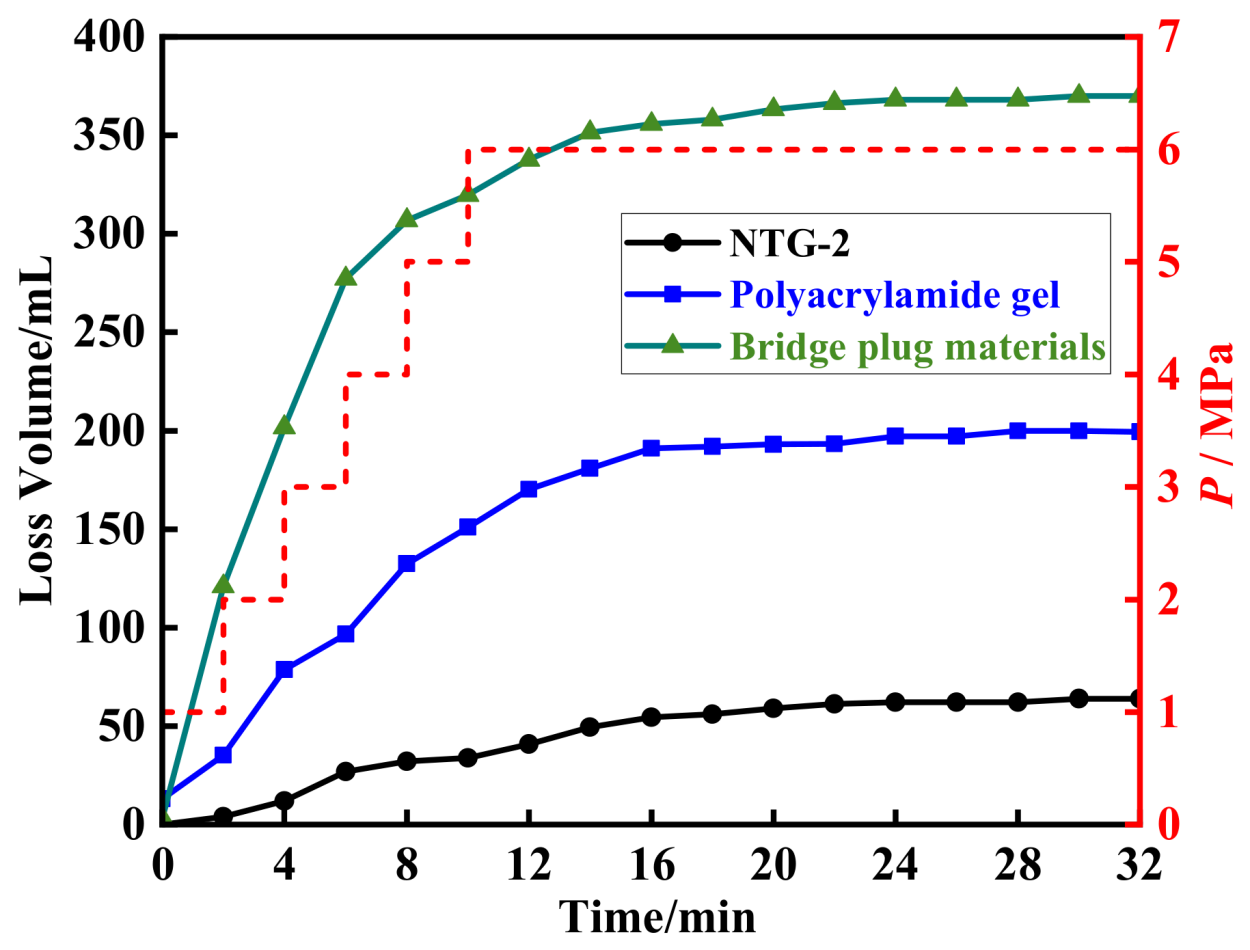
Comparison of sealing performance between NTG gel and conventional sealing agents. The red line shows the increase in pressure over time.

To evaluate the plugging performance, the dried NTG gel was crushed and sieved, and 20–80 mesh gel particles were incorporated into a bentonite-based slurry at a concentration of 2 wt%. A 40–60 mesh sand bed was used to simulate a loss channel, and the sealing performance was evaluated at 150 °C and 6 MPa. As shown in Figure 8, the NTG gel exhibited excellent plugging performance, with a cumulative fluid loss of only 63.9 mL. In comparison, the cumulative leakage of traditional polyacrylamide gel and bridging materials are 369.3 mL and 198.4 mL, respectively, demonstrating significantly superior sealing performance.

The superior plugging performance of NTG primarily results from the synergistic effects of shear thinning, water-induced swelling, and structural recovery. Under pressure, the gel reduces flow resistance and penetrates the sand-bed pores, followed by water swelling and network recovery that promote pore filling and retention. Its relatively high gel strength and viscoelasticity further maintain the integrity of the sealing structure, enabling effective fluid-loss control. In comparison, conventional polyacrylamide gels are susceptible to structural deterioration under elevated temperature and pressure, whereas bridging materials mainly rely on particle–pore size matching and have limited adaptability to the pore structure. Consequently, NTG exhibited lower cumulative fluid loss and superior overall plugging performance.

### 2.7. Mechanism Analysis

**Figure 9:**
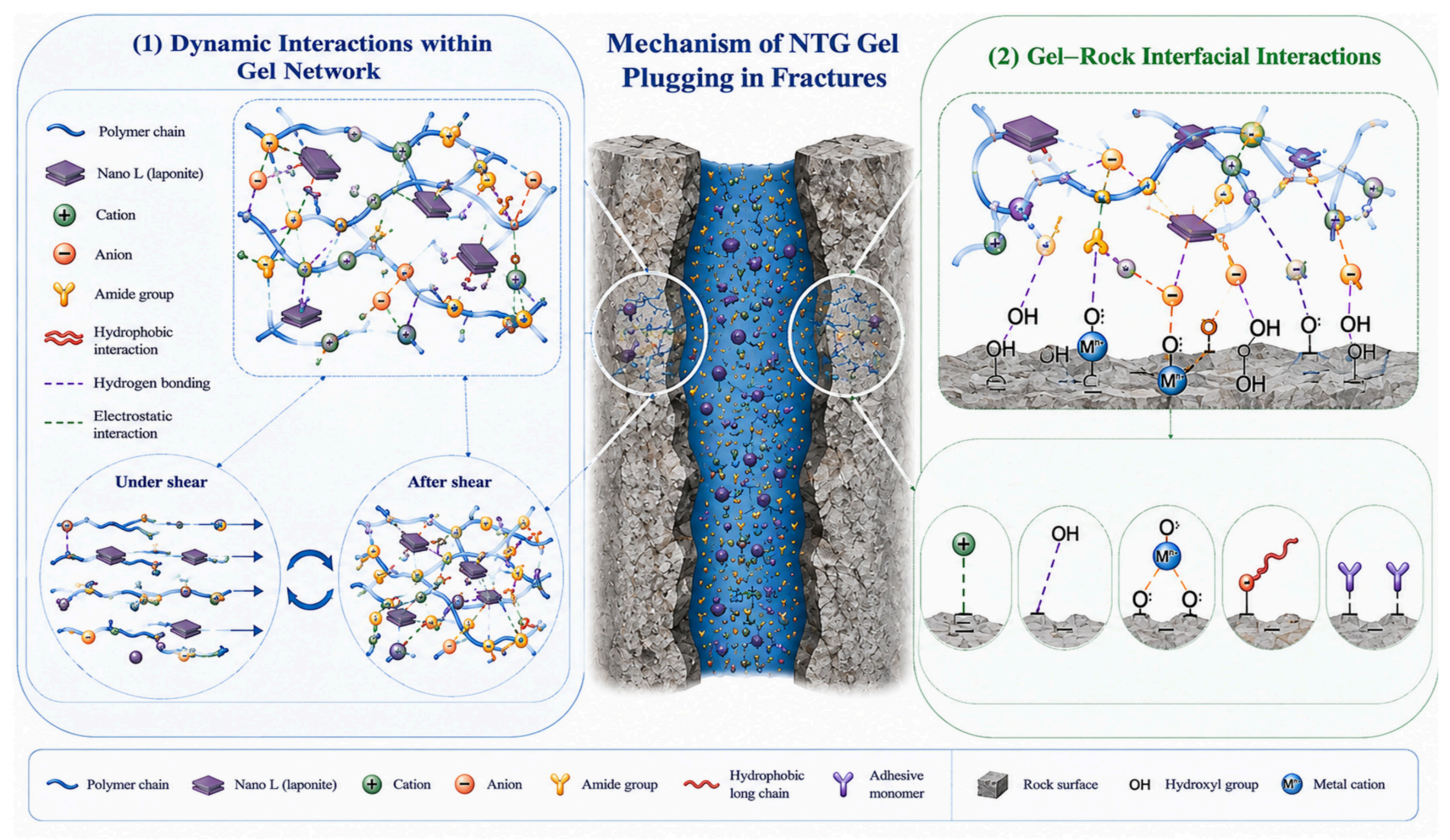
Action mechanism diagram of NTG.

The plugging mechanism of the gel within fractures mainly involves dynamic interactions within the gel network and interfacial interactions between the gel and the rock surface (Figure 9). The synergistic effects of these interactions endow the gel with favorable flow adaptability, structural recovery, and interfacial adhesion, allowing it to maintain good flowability during pumping while rapidly recovering its network structure and forming a stable sealing layer after entering the fractures.

Interactions within the gel network. The structural stability of the gel mainly originates from a dynamic crosslinked network constructed through multiple non-covalent interactions, resulting in a three-dimensional structure with a certain degree of reversibility. Under external shear, some dynamic crosslinking points undergo temporary dissociation, leading to partial disruption of the gel network, while the polymer chains become oriented along the flow direction. This reduces the flow resistance of the system, thereby imparting pronounced shear-thinning behavior and favorable pumpability to the gel. After the cessation of shear, the synergistic interactions among amide groups, ionic groups, hydrophobic long chains, adhesive monomers, and nanoparticles promote the reconnection of the network structure. Driven collectively by non-covalent interactions such as electrostatic attraction, hydrophobic association, and hydrogen bonding, interactions between molecular chains are re-established, and the dynamic crosslinking points progressively recover. Consequently, the gel network rapidly reorganizes and transforms from a flowing state into a continuous bulk gel state. This structural recovery facilitates the rapid spreading and filling of the gel within fractures and promotes the formation of a continuous and compact sealing layer, thereby improving its plugging capability against loss channels. The introduced nanoscale lithium magnesium silicate material L serves as a nano-reinforcing unit in the gel network. Through physical entanglement and multipoint hydrogen bonding with the polymer chains, it enhances the connectivity and stability of the network structure. The multiscale synergistic effect between the nanosheets and the polymer chains restricts excessive molecular chain movement and promotes network structure recovery after shearing, thereby further enhancing the thixotropic recovery capability and structural stability of the gel.

Gel–rock interfacial interactions. In addition to the dynamic network interactions within the gel, interfacial interactions between the gel and rock surface are another important factor contributing to the formation of a stable sealing structure. Active groups such as amide and catechol groups in the gel can interact with surface hydroxyl groups and metal cations on the rock surface through hydrogen bonding and metal coordination, thereby enhancing the adsorption of gel molecules and their interfacial bonding with the rock surface. In addition, the cationic groups within the gel can be electrostatically adsorbed onto the generally negatively charged rock surface, thereby enhancing gel retention within the loss channels and reducing its migration under pressure. Meanwhile, the hydrophobic long chains can interact with hydrophobic domains on the rock surface, further lowering the interfacial energy of the gel–rock interface and promoting the adsorption of adhesive monomers onto the rock surface. The synergistic effects of these interfacial interactions strengthen the interfacial bonding between the gel and rock, enabling the gel to remain stably retained along the fracture walls and, together with its three-dimensional network, form a sealing structure with high integrity and pressure-bearing capacity.

## 3. Conclusions

This study presents a nanomaterial L-modified thermally expandable thixotropic gel system designed for high-temperature fractured formations. By introducing multiple non-covalent interactions, the gel network is effectively reinforced, enabling superior rheological regulation and structural stability. Through systematic rheological and performance evaluations, the formulation was optimized to achieve a balanced combination of shear-thinning behavior, structural recovery, and plugging efficiency. The developed gel exhibits excellent injectability under high shear, maintains strong structural integrity under low shear, and demonstrates stable and effective plugging performance across varying temperatures and fracture scales.

The developed gel-based plugging system significantly enhances the adaptability of conventional materials in complex formations, offering an efficient and robust solution for lost circulation control. The findings confirm the effectiveness of nanomaterial regulation and multiscale network construction in improving gel performance while providing new theoretical insights into the design of thermally expandable plugging materials. This work expands the application potential of nanogels under extreme conditions and lays a solid foundation for the development of advanced plugging materials, with promising implications for improving operational safety and efficiency in oil and gas exploitation.

## 4. Materials and Methods

### 4.1. Materials

2-Acrylamido-2-methyl-1-propanesulfonic acid (AMPS), acrylamide (AM), acrylic acid (AA), Crosslinking agent octadecyl methacrylate (SMA), Laponite (layered magnesium–lithium silicate) nanomaterial L, Chitosan (CS), Cetyltrimethylammonium chloride (CTAC), N-Cetyl-dimethylamine, Acrylamide, dopamine hydrochloride (DA), sodium tetracetate decahydrate, hydrochloric acid, ethyl acetate, phenolic resin crosslinking agent, potassium persulfate (KPS), and nanomaterial L are all of analytical grade. Sodium hydroxide, bentonite (BNT), and sodium chloride are all industrial-grade products. Deionized water was prepared in-house. All the above medications were purchased from Shanghai Aladdin Biochemical Technology Co., Ltd. (Shanghai China).

### 4.2. Synthesis

Adding N-hexadecyl dimethylamine to ethanol, followed by the addition of acryloyl chloride, and heating under reflux; unreacted material and solvent are removed by azeotropic distillation. The product is dried by recrystallization from anhydrous ethanol to yield the hydrophobic monomer (Hy). The dopamine derivative (Do) was prepared by dissolving sodium tetraborate decahydrate, dopamine hydrochloride (DA), K_2_CO_3_, and acryloyl chloride in deoxy methanol to form a basic reaction system. The mixture was extracted with ethyl acetate; the organic phases were combined, dried over anhydrous sodium sulfate, and concentrated, Finally, Laponite (layered magnesium–lithium silicate) nanomaterial L (Modified lithium magnesium silicate), which possesses strong thixotropic properties, was added in varying proportions to thixotropic gels prepared by crosslinking acrylamide polymers with phenolic resin crosslinking agents. The resulting gel systems were named NTG-0, NTG-1, NTG-2, and NTG-3, respectively.

### 4.3. Rheological Properties Test

The shear-thinning behavior and yield stress of the gel systems were evaluated based on apparent viscosity–shear rate and shear stress–shear rate profiles. All rheological measurements were conducted at 25 °C in controlled shear rate mode, with the shear rate progressively decreased from 1000 to 1 s^−1^ over 80 measurement intervals, and each shear rate was maintained for 3 s. Accurate determination of rheological parameters and selection of appropriate constitutive models are essential for optimizing drilling parameters and improving operational efficiency. Common rheological models for drilling fluids include the Bingham plastic, power-law, and Herschel–Bulkley models, which are applicable to different types of fluid behavior. Among these models, the Herschel–Bulkley model incorporates both yield stress and nonlinear shear response and therefore provides a more accurate description of the actual rheological behavior of the gel systems. Accordingly, the Herschel–Bulkley model was employed to fit the experimental rheological data, enabling systematic comparison of the rheological parameters and their variations among different gel systems.

To comprehensively evaluate the thixotropic behavior of the gel systems, both thixotropic loop tests and multistep thixotropy tests were conducted to assess structural breakdown and recovery characteristics. In the thixotropic loop test, the shear rate was increased from 1 s^−1^ to 1000 s^−1^, held for 2 min, and then decreased back to 1 s^−1^, following a 5 min–2 min–5 min protocol. Due to the thixotropic nature of the material, a hysteresis loop is formed between the upward and downward curves, and its area reflects the extent of structural breakdown and the intensity of thixotropic behavior during shear. This method enables rapid identification of thixotropic characteristics and their relative intensity but does not provide a quantitative assessment of structural recovery. To address this limitation, a multistep strain test was further introduced, applying a low–high–low strain sequence (1%–400%–1%, each stage lasting 3 min) to simulate field conditions, including pre-injection rest, high shear during injection, and post-injection structural recovery. The structural recovery capability was quantitatively evaluated by comparing the storage modulus (G′) in the recovery stage with its initial value. All of the aforementioned rheological and thixotropy measurements were conducted at 25 °C.

### 4.4. Gelation Behavior and Mechanical Strength Test

Viscoelastic moduli of the gel systems were measured using a Haake rheometer (Thermo Fisher Scientific, Karlsruhe, Germany) to quantitatively evaluate gel strength. The measurements included stress sweep and frequency sweep tests. In the stress sweep, the frequency was fixed at 1 Hz, and the linear viscoelastic region was determined over a stress range of 1–1000 Pa. In the frequency sweep, the stress was fixed at 10 Pa, and the storage modulus (G′) and loss modulus (G″) were recorded over a frequency range of 0.1–100 Hz. All rheological measurements, including viscoelasticity, shear-thinning, and thixotropic behavior, were performed using a cone–plate geometry. All viscoelasticity, shear-thinning, and thixotropy measurements were performed using a cone–plate geometry at 25 °C to ensure consistent testing conditions among the different gel systems.

The temperature-dependent stability and swelling behavior of the gel systems were further evaluated under elevated-temperature conditions, as described in the subsequent sections.

### 4.5. Thermal Stability Test

A total of 20 g of the NTG thixotropic gel was aged at different temperatures in an oven for 24 h. The thermal stability was evaluated by measuring the mass retention and dehydration volume after aging.

### 4.6. Salt Tolerance Test

The salinity resistance of the thixotropic gel system was evaluated by measuring changes in its viscoelastic moduli using a Haake rheometer. Specifically, gel systems were prepared using NaCl solutions with different mass fractions, and their viscoelastic moduli and cyclic shear recovery behavior after gelation were measured to assess salt tolerance.

### 4.7. Plugging Performance Test

Prior to testing, the injection lines were thoroughly cleaned, and an artificial fractured core was placed in a core holder under an axial pressure of 2 MPa to ensure uniform pore structure. The prepared thixotropic gel was then injected into the sand-packed tube until the effluent was entirely replaced by the gel, indicating completion of injection. The system was subsequently heated to the target temperature and maintained for 6 h to allow gelation. After gelation, the core holder was connected to the injection system, and standard brine was injected to conduct displacement tests. The pressure evolution during displacement was recorded, and the breakthrough pressure was defined as the pressure at which a sudden drop occurred, indicating fluid penetration through the gel at a given fracture width.

### 4.8. Sealing Performance Test

A total of 100 g of quartz sand was placed at the bottom of the drilling-fluid cup to simulate a loss zone. Subsequently, 400 mL of the prepared plugging slurry was added to the cup. After the system was heated to the designated temperature and stabilized, the upper and lower valves were opened, and the pressure differential between the two valves was gradually increased to 6 MPa at a rate of 1 MPa/2 min. The fluid loss volume during this stage was recorded as V_1_. The upper and lower valves were then closed, and the pressure in the cup was reduced to 1 MPa and maintained for 6 h to simulate the residence and sealing process of the plugging material within the loss channel. After the static period, the upper and lower valves were reopened, and the pressure differential was again increased to 6 MPa at a rate of 1 MPa/2 min. The fluid loss volume during the second pressurization stage was recorded as V_2_. The cumulative fluid loss volume, V, was calculated as V = V_1_ + V_2_.

## Figures and Tables

**Table 1:** Rheological model fitting results of gel system.

Model	Parameter	NTG-0	NTG-1	NTG-2	NTG-3
Herschel–Bulkley model	τ_0_	12.66	14.57	20.05	30.23
	*K*	0.61	2.23	6.21	14.23
	*n*	0.70	0.65	0.54	0.45
	R^2^	0.971	0.992	0.993	0.990

## Data Availability

The original contributions presented in this study are included in the article. Further inquiries can be directed to the corresponding author.
